# *TP53* codon 72 polymorphism may predict early tumour progression in paediatric pilocytic astrocytoma

**DOI:** 10.18632/oncotarget.10295

**Published:** 2016-06-25

**Authors:** Samantha Mascelli, Paolo Nozza, David T.W. Jones, Carole Colin, Angela Pistorio, Claudia Milanaccio, Marcello Ravegnani, Alessandro Consales, Olaf Witt, Giovanni Morana, Armando Cama, Valeria Capra, Roberto Biassoni, Stefan M. Pfister, Dominique Figarella-Branger, Maria Luisa Garrè, Alessandro Raso

**Affiliations:** ^1^ Dipartimento Testa, Collo e Neuroscienze, Istituto Giannina Gaslini, 16147 Genoa, Italy; ^2^ UOC Anatomia Patologica, Istituto Giannina Gaslini, 16147 Genoa, Italy; ^3^ Division of Paediatric Neurooncology, German Cancer Research Center (DKFZ), Heidelberg 69120, Germany; ^4^ CRO2 UMR_S911, Inserm, Aix-Marseille Université, 13385 Marseille, France; ^5^ Epidemiologia, Biostatistica e Comitati, Istituto Giannina Gaslini, 16147 Genoa, Italy; ^6^ Department of Paediatric Oncology, Haematology and Immunology, University of Heidelberg, Heidelberg, 69120 Heidelberg, Germany; ^7^ Neuroradiologia Istituto Giannina Gaslini, 16147 Genoa, Italy; ^8^ Laboratory Molecular Medicine, Translational Medicine Department, Istituto Giannina Gaslini, 16147 Genoa, Italy; ^9^ APHM, Hôpital de la Timone, Service d'Anatomie Pathologique et de Neuropathologie, 13385 Marseille, France; ^10^ Centro di Neuro-Oncologia, Istituto Giannina Gaslini, 16147 Genoa, Italy

**Keywords:** low-grade gliomas, polymorphism, pilocytic astrocytoma, paediatric, TP53

## Abstract

Pilocytic astrocytoma and ganglioglioma may occur in inaccessible or surgically difficult areas. In case of incomplete resection, the availability of biological predictors of tumour progression could be particularly important. To this end, an analysis of p53 codon 72 polymorphism and assessment of its role as prognostic marker were performed.

The status of the p53 Arg72Pro polymorphism was evaluated by pyrosequencing method in a multicenter cohort of 170 paediatric patients. Genotype/phenotype associations were investigated either by means of bivariate or multivariate analyses.

In the partially resected pilocytic astrocytomas, the Arg/Arg variant predicts early tumour progression (median survival time: 23.1 months) and is associated with poor event-free survival (*p* value = 0.0009). This finding remains true also in case of adjuvant therapies, with a 5-year event-free survival of 30.6% for cases with Arg/Arg variant vs. 78.7% for those with other genotypes. There is no association between ganglioglioma and the polymorphism.

The assessment of Arg/Arg variant could improve the management of pilocytic astrocytoma. *TP53* codon 72 analysis could distinguish low-risk cases, in which surgery could be conservative, from high-risk cases needing an aggressive surgery plan.

## INTRODUCTION

Genetic studies suggest that some functional single-nucleotide polymorphisms (SNPs) involved in cell death control and DNA-repair mechanisms influence the prognosis of sporadic tumours. In other words, SNPs could act on either the regulation of apoptotic potential or the maintenance of genomic stability through different DNA repair pathways [1, 2]. An example is the p53 protein, which responds to a variety of cellular stresses performing multiple functions involved in cell cycle control [3]. Notably, p53 function is perturbed by DNA sequence variations, such as SNPs. To date, several *TP53* SNPs have been characterized, many of them being localized in non-coding regions of the gene [4]. Among those found in the *TP53* coding region, the variant at codon 72 (rs1042522) is the most extensively studied. This consists of a G/C variation resulting in a non-conservative change of an arginine (R) to a proline (P) in exon 4 (Arg72Pro;). Residue 72 is located within a proline-rich region and may affect the structure of the putative SH3-binding motif (PXXP), one of the p53 DNA-binding motifs responsible for the pro-apoptotic function of the protein [3]. The Arg72 isoform is more efficient in inducing apoptosis, while the Pro72 variant activates transcriptionally several p53-dependent genes involved in DNA-repair and cell-cycle arrest [5]. A possible association between *TP53* codon 72 variants and cancer progression has been reported for several tumour types [6–15].

Low-grade gliomas (LGGs) and glioneuronal tumours are the most common brain tumours in childhood and adolescence. Among them, pilocytic astrocytomas (PAs) are the predominant pathological subtype, followed by mixed neuronal and glial tumours, such as gangliogliomas (GGs) and desmoplastic infantile gangliogliomas (DIGs). LGGs generally growth slowly and show favourable outcome, although 10–20% of children develop progressive disease or recur [16–18]. The extent of tumour resection remains the main prognostic factor: after complete surgical resection 10- year overall survival rates are 90% or higher [19]. Conversely, whenever complete resection is not achieved, either because of anatomical location or metastatic disease, adjuvant therapies, including further surgery, chemotherapy and radiation, could be necessary [17, 20–22]. As yet, neither biological predictors nor the temporal window critical for early detection of progression are known. These tumours may progress early, within 2 years from diagnosis or become stable for more than 5 years. As a consequence, there is a clinical need to find molecular predictors for early progression in paediatric LGGs and glioneuronal tumours as well as for long-term outcome. Despite *TP53* inactivation in several tumours, [23–25] somatic mutations are rare in paediatric LGGs [26–28]. It is noteworthy, however, that few studies addressed the role of Arg72Pro SNP in glial tumours: most of them included high grade tumours or adult cases and yield inconsistent results [6, 15, 29–30].

Here, we focused on the p53 Arg72Pro status in a multicenter cohort of paediatric PAs and WHO grade I mixed neuronal and glial tumours, testing its role both as risk factor and marker of early progression.

## RESULTS

### p53 codon 72 distribution in the cohort

The genetic analysis of p53 codon 72 was performed on 170 tumours from patients affected by PAs and mixed neuronal and glial tumours (GGs and DIGs). The genotype distribution of the SNP was the following: Arg/Arg 48.2% (*n* = 82); Arg/Pro 42.4% (*n* = 72); Pro/Pro 9.4% (*n* = 16), and are reported in Table 1. The distribution of both genotype and allele frequencies in all patients met the Hardy-Weinberg Equilibrium.

**Table 1:** Distribution of p53 (Arg72Pro) genetic variant among 170 cases and its association with clinical and genetic characteristics

		No. (%) per genotype					No. (%) per allele		
		No.	Arg/Arg*	Arg/Pro*	Pro/Pro*	*p* value	Arg*	Pro*	*p* value
	Patients	170	82 (48.2)	72 (42.4)	16 (9.4)	0.38^a^	236 (69.4)	104 (30.6)	0.17^a^
	Controls	192	104 (54.2)	76 (39.6)	12 (6.2)		284 (74.0)	100 (26.0)	
									
Patient Gender:	male	88	44 (50.0)	40 (45.5)	4 (4.5)	0.08^a^	128 (72.7)	48 (27.3)	0.17^a^
	female	82	38 (46.3)	32 (39.0)	12 (14.6)		108 (65.9)	56 (34.1)	
Histology:	Mixed neuronal and glial tumors	31	16 (51.6)	11 (35.5)	4 (13)	0.59^b^	43 (69.3)	19 (30.6)	0.99^a^
	Pilocytic Astrocytoma	139	66 (47.5)	61 (43.9)	12 (8.6)		193 (69.4)	85 (30.6)	
Age at diagnosis:	≤ 24 months	24	16 (66.7)	5 (20.8)	3 (12.5)	0.05^b^	37 (77.1)	11 (22.9)	0.21^a^
	> 24 months	146	66 (45.2)	67 (45.9)	13 (8.9)		199 (68.2)	93 (31.8)	
Brain lesion site:	Supratentorial	65	34 (52.3)	24 (36.9)	7 (10.8)	0.52^a^	92 (70.8)	38 (29.2)	0.67^a^
	Infratentorial	105	48 (45.7)	48 (45.7)	9 (8.6)		144 (68.6)	66 (31.4)	
EFS:	progression	26	13 (50.0)	10 (38.5)	3 (11.5)	0.82^b^	36 (69.2)	16 (30.8)	0.98^a^
	stable disease	144	69 (47.9)	62 (43.1)	13 (9.0)		200 (69.4)	88 (30.6)	
BRAF, V600E	mutation	19	9 (47.4)	6 (31.6)	4 (21.0)	0.27^b^	24 (63.2)	14 (36.8)	0.37^a^
	Wild-type	115	57 (49.6)	48 (41.7)	10 (8.7)		16 (70.4)	68 (29.6)	
KIAA1549-BRAF gene fusion	Present	84	42 (50.0)	34 (40.5)	8 (9.5)	0.76^b^	118 (70.2)	50 (29.8)	0.97^a^
	Absent	40	21 (52.5)	14 (35.0)	5 (12.5)		56 (70.0)	24 (30.0)	

* p53 codon 72;

a *P* values: Pearson *χ*^2^ test;

b *P* values: Fisher's Exact test.

Congruent results were obtained from the analysis on somatic tumour DNAs and germ-line DNAs from matched blood samples: no difference in p53 codon 72 variants was seen between isogenic samples. The bivariate analyses through cases stratification by the main histological and clinical features revealed no differences in the distribution of genotype and allele frequencies. Similarly, no relationship was found with *KIAA1549-BRAF* gene-fusion and *BRAF* mutations (Table 1).

### Arg/Arg72 p53 variant correlates with poor EFS for partially resected PAs

The Incidence Rates (IR) with selected clinical features were evaluated for PAs and mixed neuronal and glial tumours separately. The analysis on PAs (*n* = 138) did not show any association between p53 codon 72 SNP distribution and EFS when extent of resection was not taken into account, as shown in Supplementary Table 1.

**Figure 1:**
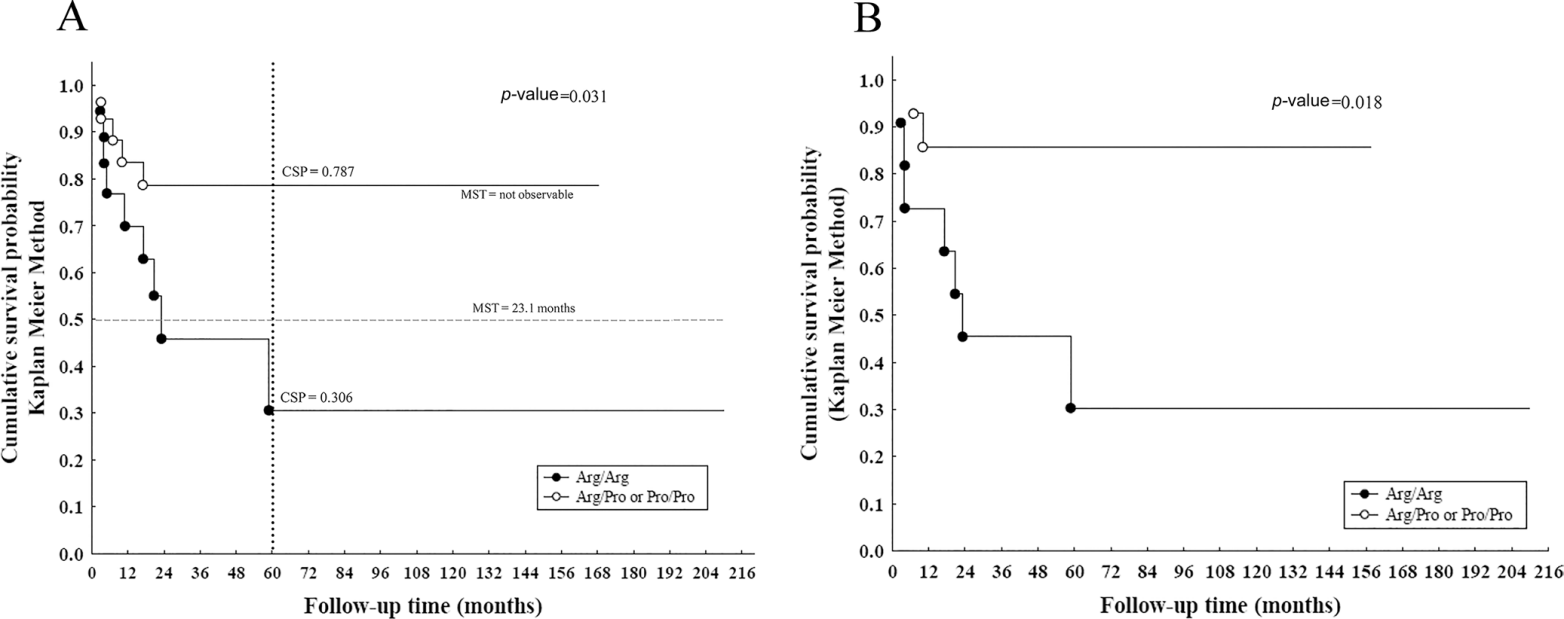
Survival curves of PAs in case of incomplete resection (**A**) Kaplan-Meier survival curve of EFS for only PAs (*n* = 46) showed shorter survival in cases with Arg/Arg variant with a cumulative survival probability (CSP) of 30.6% respect to 78.7% in the other genotypes containing Proline and, with a MST of 23.1 months, a predictor of early progression events (Log-rank test, *p* value = 0.031); (**B**) EFS for PA with additional treatments (*n* = 26) showed shorter survival in presence of Arg/Arg variant (Log rank test, *p* value = 0.018) compared with the other genotypes.

When only partially or sub-totally resected (STR) tumours were considered (*n* = 46), the incidence of progressions was significantly higher in tumours harbouring Arg/Arg variant (14.77 × *1000 p-m*) compared to those harbouring Arg/Pro or Pro/Pro variants (3.78 × *1000 p-m*) in the dominant model (*p* value = 0.031) (Table 2). Patients with Arg/Arg genotype showed a significantly worse EFS than those with a Proline variant, with a 5-year EFS of 30.6% *vs.* 78.7%, respectively (Figure 1A). The median survival time (MST) of patients with PA who underwent STR was 23.1 months for the Arg/Arg genotype, while it was statistically not observable for the Arg/Pro or Pro/Pro genotype grouping (Figure 1A). Likewise, in presence of adjuvant therapies (*n* = 26), Arg/Arg genotype showed significantly shorter EFS compared with the other genotypes (*p* value = 0.018) (Figure 1B).

**Table 2:** Number of deaths/disease progression (and percentage) and EFS: IR for progression events in patients undergoing to STR with PA (*n* = 46) (*n* = 14/46; 30.4%), IR: 7.3 × 1000 p-m (95% CI: 4.3–12.2)

		No. of disease progression or deaths (%)	EFS IR ×1000 p-m (95% CI)	*p* value Log-Rank
Gender:	Male (*n* = 23)	3 (13.0%)	2.485 (0.8–7.7)	*0.012
	Female (*n* = 23)	11 (47.8%)	15.179 (8.4–27.4)	
Age at diagnosis:	≤ 24 months (*n* = 8)	4 (50%)	10.352 (3.9–27.6)	0.30
	>24 months (*n*= 38)	10 (26.3%)	6.469 (3.5–12.0)	
Site of lesion:	Supratentorial (*n* = 21)	8 (38.1%)	9.401 (4.7–18.8)	0.41
	Infratentorial (*n* = 25)	6 (24.0%)	5.550 (2.5–12.4)	
Additional treatments:	Yes (*n* = 25)	9 (36%)	6.800 (3.5–13.1)	0.87
	No (*n* = 21)	5 (23.8%)	8.214 (3.4–19.7)	
p53 Arg72Pro(dominant model):	Arg/Arg (*n* = 18)	9 (50.0%)	14.769 (7.7–28.4)	*0.031
	Arg/Pro or Pro/Pro (*n* = 28)	5 (17.9%)	3.780 (1.6–9.1)	

IR × 1000 p-m (95% CI): Incidence Rate × 1000 person-months (95% Confidence Intervals);

* significant *p* values

Furthermore, gender-stratified incidence rates differed significantly (*p* value = 0.012): female patients (15.18 × *1000 p-m*) had a worse EFS than male patients (2.48 × *1000 p-m*) (Table 2). Regarding age and tumour site of lesion at diagnosis, no difference in EFS was observed (*p* value = 0.30 and *p* value = 0.41, respectively).

Multivariate analysis using Poisson regression model identified Arg72Pro SNP and gender associated with risk of progression in patients with partial tumour resection. An increased risk for progression was observed in Arg/Arg genotype (adjusted IRR = 6.4, 95% CI: 2.1–19.3; *p* value = 0.0009), and in female patients (adjusted IRR = 9.4, 95% CI: 2.6–34.3; *p* value = 0.0001) (Table 3). The remaining factors tested in the model were not associated with a higher risk disease progression: age at diagnosis (*p* value = 0.76), site of lesion (*p* value = 0.36), additional treatments (*p* value *=* 0.72).

**Table 3:** Best fitted Poisson Regression model

Patients (No. of events = 14/46; 30.4%).		Hazard Ratio (HR)^a^	95% CI	*p* value
Gender:	Female *versus* Male	9.4	2.6–34.3	*0.0001
p53 Arg72Pro *(dominant model)*:	Arg/Arg *versus* Arg/Pro or Pro/Pro	6.4	2.1–19.3	*0.0009

EFS; time variable measured from diagnosis to date of progression or death (months) in patients undergoing to STR with PA (*n* = 46).

* significant *p* values.

Moreover, incidence rates evaluated only in mixed neuronal and glial tumours did not show any association with the *TP53* codon 72 SNP *(* Supplementary Table 2).

## DISCUSSION

PAs and mixed neuronal and glial tumours occurring in surgically non amenable sites pose several challenges to paediatric oncologists. Their significant morbidity is partly related to therapy side effects. Thus, it is extremely important to tailor the therapies more precisely.

We focused on the p53 Arg72Pro status in a multicenter cohort of PAs, GGs, and DIGs, testing its role both as a risk factor and biological marker of early progression. The current study is focused upon a well-defined series of paediatric cases arising in the European-Caucasian population, recruited over an 18-year period.

The biological value of p53 Arg72Pro variant in cancer risk and progression is extremely controversial [6, 7, 31]. A possible explanation could be found in population genetics. Indeed, a meta-regression study suggested that ethnicity, as well as histotyping, anatomical sites and genotyping method, are responsible for most of the heterogeneity observed [32]. Recently, *TP53* polymorphism has been investigated in adult high-grade gliomas [33]. An association between the Arg72 variant has also been reported to increase risk of developing, lymphocytic leukaemia and gastric and skin cancer [8, 34].

Herein, a cohort of European-Caucasian patients has been analyzed, since sharp ethnic differences were previously observed for Arg72Pro SNP. In fact, the Arg72 variant was found more prevalent in European-Caucasians, whereas the Pro72 variant was predominant in Chinese and African-Americans. A significant North-South gradient for the Arg allele was found, with an increase in allele frequency in association with distance from the equatorial plate [35].

Our results showed no significant difference in the genotype and allele frequencies between tumours and controls, and no significant association when tumour samples were stratified by neuropathological features (Table 1). Moreover, no difference was seen at p53 codon 72 between tumour and isogenic blood, indicating that such analysis can be carried out from either material.

Intriguingly, the survival analysis showed a statistically significant difference in EFS for European-Caucasian patients with PAs for whom a gross total resection was not achieved: EFS was worse for patients with PA carrying Arg/Arg variant than the other genotypes in a *dominant model* (Figure 1A), indicating a prognostic impact on early progression events. Interestingly, Arg variant seems to favour inactivation of p73-dependent apoptosis in tumours with mutant p53 [4, 5, 36]. Since dysfunctional missense *TP53* mutations are rare in childhood LGGs, the codon 72 SNP probably influences clinical course in combination with clinical variables or other genetic variations.

No association with *TP53* polymorphism was found in mixed neuronal and glial tumours (Supplementary Table 2). Thus, independently from resection type and site of lesion, *TP53* codon 72 SNP is not a EFS predictor marker. A deeper analysis was not possible because of the small sample size of each group.

Conversely, p53 Arg/Arg72 variant reliably predicts early progression in partially resected PAs (Table 2), also independent of the adjuvant treatment (Figure 1B). Thus, this polymorphism may even modulate individual response to therapy [6, 7]. Radiotherapy and chemotherapy cause DNA damage. This SNP, is essential for p53-induced cell cycle arrest, and, in presence of extensive DNA damage, promote the irreversible growth arrest (senescence) or induce apoptosis [7]. Glioma cells choose tumour regulation growth through via senescence [37]: our findings suggest that, in some way, the presence of Arg72 in homozygous is an adverse prognostic factor in case of tumour residual.

The relation of Arg/Arg variant to EFS has been verified in multivariate analysis (Table 3). Noteworthy, as previously reported [19], gender seems to be an independent risk factor for PA progression (Table 2, 3).

In summary, we highlighted the role of p53 Arg/Arg72 variant as a predictor for early progression in partially resected PAs. Further validation in another cohort is needed to include such a marker in future trials by guiding future p53-activation therapy. This result could significantly change the patient management, distinguishing low risk cases in which surgery could be conservative, from cases for whom a more aggressive surgery plan could be appropriate. Thus, the inclusion of this SNP analysis into molecular screening might influence the choice of adjuvant therapies or plan proper follow-up. The genetic test could be easily performed on blood samples, and would be a quick, non-invasive and cheap method.

## MATERIALS AND METHODS

### Participants

**Figure 2:**
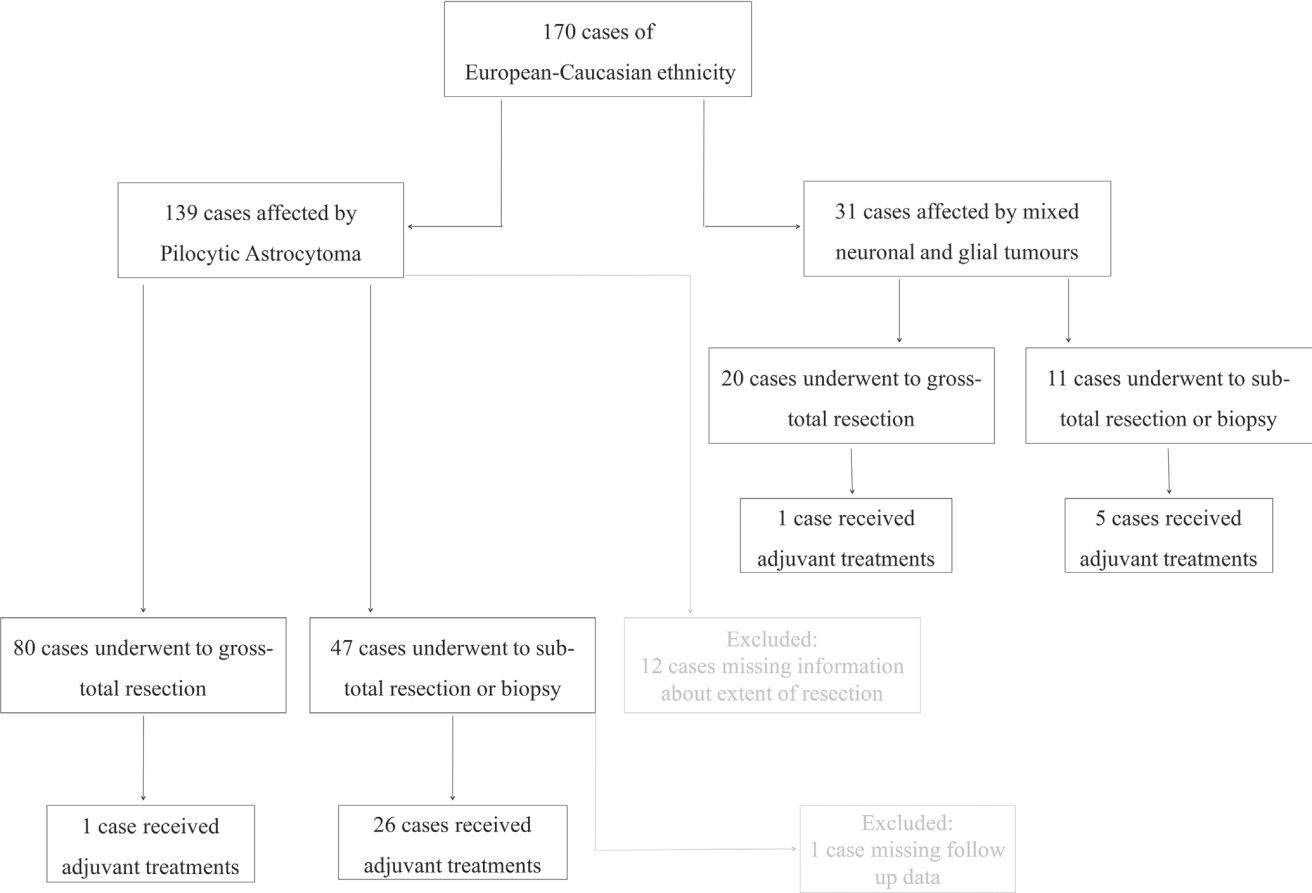
The flow chart of patient selection, including histological and extension of surgical resection criteria The excluded cases by the analysis are reported in grey colour.

The study cohort consisted of 170 tumours from patients of European-Caucasian ethnicity affected by pilocytic astrocytomas (PAs) and mixed neuronal and glial tumours (GGs and DIGs), including 66 cases treated at the Giannina Gaslini Institute, Genoa - Italy, 20 cases from the Marseille series (retrieved from the Assistance Publique- Hôpitaux de Marseille -AP-HM tumor bank, France- authorization number AC-2013-1786) and 84 cases from the Heidelberg cohort (German Cancer Research Centre –DKFZ-, Heidelberg, Germany). For all cases clinical data were available. Patients' median age at diagnosis was 7.0 years (range 4.9 months – 16.6 years) and all CNS sites were affected. Median follow-up time was 7.4 years ± 4.2 SD. Surgical treatment consisted of 100 (63.3%) complete and 58 (36.7%) partial removals; no information available in 12 cases. Progression event occurred in 25 cases (14.8%), whose 17 were PA and 8 were mixed neuronal and glial tumours. In the presence of a residual tumour tissue, an observation period or the administration of adjuvant therapies, consisting of radiotherapy and/or chemotherapy, were offered according to the International Society of Paediatric Oncology protocol for low-grade gliomas (SIOP-LGG) [38]. Clinic-pathological characteristics are listed in Supplementary Table 3, and summarized in a flow chart, as shown in Figure 2.

For the mutational screening of *TP53* codon 72 SNP, we analyzed DNA from 86 cases out of the Italian and French patients (55 PAs and 31 mixed neuronal and glial tumours): only samples with a tumour cell content of at least 80% were used for molecular screening. Conversely, genetic data of 84 PAs were directly collected from the Heidelberg files. Genetic analysis of this cohort has been described previously [39]. Furthermore, peripheral matched blood DNA from 66 patients and from 192 healthy children (median age 10 years) was also investigated.

Correlation analysis between *TP53* codon 72 variant and *BRAF* alteration were performed using previously described *BRAF* genetic data [39–41].

Written informed consent was obtained from all the patients' parents or guardians, as well as for the controls.

The experiments described comply with the current laws of the countries in which they were performed. The local Ethics Committee for human studies approved the research according to Institutional guidelines.

### TP53 codon 72 pyrosequencing assay

Genomic DNA was isolated by using the GenElute Mammalian Genomic DNA Miniprep kit (Sigma, St. Louis, MO) from both frozen sections of tumour tissue samples and peripheral blood, according to the manufacturer‘s protocol. Primers for *TP53* codon 72 variant (rs1042522) were designed following the manufacturer's protocols for pyrosequencing. The locus-specific codon 72 of *TP53* was amplified using 200 nM of TP53-F-5′-GCTGTCCCCGGACGATATT-3′ and TP53-R-5′-GCCGGTGTAGGAGCTGCTG-3′ primers, respectively. The forward primer contained biotin at the 5′ position. Amplification was carried out in a 25 micro-litre final volume reaction using 20 ng of genomic DNA. Thermal profile used was 2 minutes 94°C, and 35 cycles with 30 seconds at 94°C, 30 seconds at 58°C, and 30 seconds at 72°C, followed by 7 minutes at 72°C.

All pyrosequencing reactions were performed with a reverse sequencing primer 5′-GGTGCAGGGGCC ACG-3′ used to detect a sequence like C/GGGGGAGCAGC CTCTGGCATTCTGGG as previously described [42].

### Statistical analysis

Descriptive statistics were reported in terms of absolute frequencies or percentages for qualitative data, while in terms of medians, first and third quartiles (1st–3rd q), standard deviations (SDs), minimum, and maximum values for continuous quantitative data. Deviation from Hardy-Weinberg Equilibrium of Arg72Pro SNP was determined using a goodness of fit chi-square (χ^2^) test. Pearson's chi-square test (or Fisher's Exact test in case of expected frequencies lower than 5) was used to compare the distribution of genotype and allele frequencies among patients and controls.

Considering the clinical course of PAs and of mixed neuronal and glial tumours, EFS rather than Overall Survival (OS), was chosen as the primary end-point. Furthermore, since only two events of death were present among the cohort, data was considered not sufficiently informative to even perform OS analysis. EFS was defined as the time from diagnosis to the date of progression or date of death, and all patients surviving at the time of analyses were censored at the date of last contact.

IR of death or disease progression were calculated and reported with their 95% Confidence Intervals (95% CI). Survival curves were drawn to assess disease-free survival and time to the event was expressed as *person-months*. Survival curves were constructed according to the Kaplan-Meier method and compared by the log-rank test.

Finally, a Poisson regression model was performed in order to evaluate the role of clinic-pathological and genetic variables in influencing hazard ratios (HR). Statistically significant variables in the bivariate analysis or clinically important variables were subsequently included in the multivariate analysis. The following variables were considered into the saturated model: gender (female *vs.* male), age at diagnosis (≤ 24 months *vs.* > 24 months), site of lesion (supratentorial *vs.* infratentorial), additional treatments (yes *vs.* no) and p53 codon 72 SNP (Arg/Arg *vs.* Arg/Pro or Pro/Pro).

Likelihood Ratio test (LR test) was used for comparisons and HR with their 95% Confidence Intervals (95% CIs) were calculated and reported. All statistical tests were 2-sided, and *p* values less than 0.05 were considered to be statistically significant. The software “Statistica” (release 9.0, StatSoft Corporation, Tulsa, OK) was used for descriptive and bivariate analyses, and Stata software, version 7 (Stata), was used for survival and multivariate analyses.

## SUPPLEMENTARY MATERIALS TABLES


