# Supplementary material for: TP53 codon 72 polymorphism may predict early tumour progression in paediatric pilocytic astrocytoma
Source: Oncotarget. 2016 Jun 25;7(30):47918–26. doi: 10.18632/oncotarget.10295 (PMC5216988; doi:10.18632/oncotarget.10295)
Supplement: Supplementary file 1 [file oncotarget-07-47918-s001.pdf]

TP53 codon 72 polymorphism may predict early tumour progression in paediatric pilocytic astrocytoma

Supplementary Materials

Supplementary Table 1: Number (and percentage) of deaths or disease progression in PA in European patients (*n* = 138) (*n* = 17; 12.3%)

	No. of deaths/disease progression (%)	IR × 1000 p-m	*p* value Log-Rank
Gender: Male (*n* = 74)	5 (6.8%)	1.267 (0.527–3.043)	0.031
Female (*n* = 64)	12 (18.7%)	3.977 (2.259–7.003)	
Age at diagnosis: ≤ 24 months (*n* = 15)	4(26.7%)	4.658 (1.748–12.412)	0.098
> 24 months (*n* = 123)	13 (10.6%)	2.129 (1.236–3.667)	
Site of lesion: Supratentorial (*n* = 40)	8 (20.0%)	3.422 (1.711–6.843)	0.150
Infratentorial (*n* = 98)	9 (9.2)	1.945 (1.012–3.738)	
Extent of resection: Subtotal resection (STR) (*n* = 46)	14 (30.4%)	7.246 (4.291–12.234)	*0.0001
Gross total resection (GTR) (*n* = 80)	3 (3.7%)	0.889 (0.287–2.758)	
p53 Arg72Pro, (dominant model): Arg/Arg (*n* = 65)	9 (13.8%)	2.922 (1.520–5.616)	0.469
Arg/Pro or Pro/Pro (*n* = 73)	8 (11.0%)	2.059 (1.030–4.118)	

*significant *p* values

Supplementary Table 2: Number of deaths/disease progression (and percentage) and EFS: IR for progression in mixed neuronal and glial tumors (*n* = 11) who underwent to STR with selected clinical features, in correlation with p53 Arg72Pro SNP

	No. of deaths/disease progression (%)	EFS IR × 1000 p-m	*p* value Log-Rank
p53 Arg72Pro *(dominant model):* Arg/Arg (*n* = 5)	2 (40.0)	6.282 (1.571–25.118)	0.30
Arg/Pro or Pro/Pro (*n* = 6)	3 (50.0)	10.341 (3.335–32.062)	

IR × 1000 p-m: Incidence Rate × 1000 person-months; *significant *p* values

Supplementary Table 3: Clinicopathological data of the cohort (*n* = 170)

**Characteristic**			
Median age (years) - (1st-3rd q)	7.0 (3.0–11.0)		
Age ≤ 24 months: –No. – (%)	24 (14.1%)		
Median (1st-3rd q) of follow-up time (years):	2.7 (0.8–8.4)		
		**No.**	**%**
Gender	Male	88	51.8
	Female	82	48.2
Histology	Pilocytic astrocytomas	139	81.8
	Mixed neuronal and glial tumours	31	18.2
Site of lesion	Cerebellum	65	38.2
	Cerebral hemisphere	35	20.6
	Optic pathway/hypothalamus	19	11.2
	Thalamus/mesencephalic	12	7.1
	Brainstem/spinal cord	26	15.3
	4th ventricle	5	2.9
	Cerebellum peduncle	2	1.2
	Unspecified posterior fossa	6	3.5
Extent of resectiona	Gross total resection (GTR)	100	63.3
	Partial resection or biopsy (STR)	58	36.7
Adjuvant treatment b	yes	33	19.4
EFS	With stable disease	144/169	85.2
	disease progression	25/169	14.8

[a]for 12 case surgical information is not available; [b]adjuvant therapies information is missing for 21 cases.
